# The high-pathogenicity island (HPI) promotes flagellum-mediated motility in extraintestinal pathogenic *Escherichia coli*

**DOI:** 10.1371/journal.pone.0183950

**Published:** 2017-10-10

**Authors:** Giuseppe Magistro, Christiane Magistro, Christian G. Stief, Sören Schubert

**Affiliations:** 1 Department of Urology, Ludwig-Maximilians-Universität, Munich, Germany; 2 Max von Pettenkofer-Institut für Hygiene und Medizinische Mikrobiologie, Munich, Germany; Beijing Institute of Microbiology and Epidemiology, CHINA

## Abstract

The key of success of extraintestinal pathogenic *Escherichia coli* (ExPEC) to colonize niches outside the intestinal tract and to establish infection is the coordinated action of numerous virulence and fitness factors. The so-called high-pathogenicity island (HPI), responsible for synthesis, secretion and uptake of the siderophore yersiniabactin, proved to be an important virulence determinant. In this study we investigated the interaction of the flagellum-mediated motility and the HPI. The impairment of yersiniabactin production by deletion of *irp2* or *ybtA* affected significantly motility. The gain of yersiniabactin production improved motility in both pathogenic and non-pathogenic *E*. *coli* strains. The loss of flagella expression had no adverse effect on the HPI. Strikingly, external iron abundance was not able to suppress activation of the HPI during motility. The HPI activity of swarming bacteria was comparable to iron deplete conditions, and could even be maximized by supplementing excessive iron. This fact is the first description of a regulatory mechanism, which does not follow the known hierarchical regulation of siderophore systems. Transcriptional reporter fusions of the *ybtA* promoter demonstrated that the entire promoter region with all YbtA binding sites is necessary for complete induction in both HPI-positive and HPI-negative strains. Altogether, these results suggest that the HPI is part of a complex regulatory network, which orchestrates various virulence mechanisms to optimize the overall fitness of ExPEC.

## Introduction

Over the last decades the growing body of evidence was helpful to elucidate the pathogenic potential of extraintestinal pathogenic *E*. *coli* (ExPEC) [1–3]. The orchestrated action of a plethora of virulence and fitness factors enables ExPEC to colonize and to establish infections outside the intestinal tract resulting in diseases like urinary tract infection (UTI), neonatal meningitis, sepsis, intraabdominal infection, pneumonia, osteomyelitis, cellulitis and wound infection. Transcriptomic and proteomic approaches were performed to identify determinants essential to the pathogenesis of UTI [4–6]. It is striking to note that iron acquisition systems always proved to be key players. To face the iron scarcity of the urinary tract, ExPEC has evolved a multi-factorial strategy to scavenge efficiently for this nutrient [7]. The so-called high pathogenicity island (HPI), responsible for synthesis, secretion and uptake of the siderophore yersiniabactin, represents one of these fundamental iron uptake systems. This pathogenicity island was firstly described in *Yersinia* spp. and spread in a big bang-like moment over a variety of *Enterobacteriaceae*, where it plays a major role in virulence [8–10]. As a pathogenicity island it displays typical features [11–14]: (i) a gene cluster large in size (≥35kb); (ii) location next to a tRNA encoding gene; (iii) a G+C content higher than the host chromosome; (iv) it carries a gene coding for an integrase; (v) the final product contributes to virulence. The genetic organization and regulation of the HPI have been subject of intense research [15]. The mixed-type siderophore yersiniabactin is synthesized by a mixed nonribosomal peptide synthetase (NRPS) / polyketide synthase (PKS) process [16]. YbtA, a transcriptional regulator of the AraC-like family, and the iron master regulator Fur control the transcriptional regulation of the four operons located within the HPI, (i) *ybtA*, (ii) *fyuA*, (iii) *irp2- irp5*, (iv) *irp6-irp9* [17–19]. The promoters of *irp2*, *fyuA*, and the divergent overlapping promoter region between *irp6* and *ybtA* contain specific binding sites termed repeated sequences (RSs). YbtA is proposed to bind to these sites as a homodimeric complex with yersiniabactin. Full expression of Irp2, FyuA and Irp6 depend on the action of YbtA. Here the transcriptional regulator works as an activator. Regarding its own transcription, YbtA shows auto-repression.

Interestingly, the supply of ferric iron to the microbial cell is considered to be the main function, but recent studies indicate the implication of the HPI in various processes apart from just iron acquisition. So Paauw et al. reported that the high binding affinity of yersiniabactin for ferric iron not only promotes bacterial growth by supplying additional iron but also reduces the production of reactive oxygen species by activated immune cells [20]. Furthermore, elements expressed by the HPI for the uptake of ferric yersiniabactin display additional functions. So, the outer membrane receptor FyuA contributes to efficient biofilm formation in human urine and deletion of *fyuA* additionally led to morphological changes of bacteria during biofilm maturation [21]. With FyuA being pathogen-specific, antigenic, surface exposed and *in vivo* expressed it fulfils all essential criteria of a potential vaccine candidate [22]. A multiepitope vaccine containing immunodominant epitopes of iron uptake receptors including FyuA was developed and conveyed protection against ExPEC in a murine model of peritonitis [23; 24]. Another study investigating the primary metabolome of uropathogenic *E*. *coli* (UPEC) strain UTI89 during growth in minimal medium revealed metabolic changes when genes of the HPI were mutated [25]. An extraordinary observation regarding the HPI function has been reported in UPEC strain CFT073 [26]. This isolate is unable to produce yersiniabactin due to mutations of biosynthetic genes [27; 28]. The fact gives reason to believe, that the deletion of the entire genomic island harbouring the HPI would have no impact on the pathogenicity in a murine model of ascending UTI. Most strikingly, in a co-challenge infection with the wild type strain, the deletion mutant demonstrated a log–scale reduction in colonization of the kidneys.

The versatility of the HPI clearly shows how this acquired iron uptake system became part of a complex network that coordinates various virulence and fitness properties. This multi-functional aspect prompted us to investigate whether additional virulence mechanisms may utilize the HPI. The hostile environment of the urinary tract forces ExPEC to get access to more favourable sites to scavenge for nutrients like iron, as well as to escape the host immune response. In this regard, flagellum-mediated motility has been demonstrated to be of great importance [29–31]. Only a few studies tried to relate the role of siderophore mediated iron uptake to motility. For example, the loss of pyoverdine synthesis in *Pseudomonas putida* abolished completely bacterial surface movement [32]. A functional genomic approach with swarming *Salmonella typhimurium* revealed a strong induction of iron uptake systems during motility [33]. In the case of *Vibrio parahaemolyticus*, it is known that iron depletion is an essential signal for swarmer cell differentiation [34]. A relevant role of iron homeostasis for motility has also been reported in UPEC [35]. The iron master regulator Fur represses iron uptake systems by binding to specific Fur binding-sites, so called Fur boxes, in complex with ferrous iron under iron abundance. Whenever iron is limited, Fur is inactivated and Fur-regulated genes are de-repressed. Fur boxes were also identified upstream of the activator of flagellar expression *flhD*. Both deletion of *fur* and iron scarcity had a strong impact on motility. Altogether, these examples point towards an important contribution of iron acquisition systems to flagellum-mediated motility. In this work, the main objective is to examine the interaction of the HPI and motility in ExPEC. We present data showing the strong induction of the HPI in motile bacteria and the impact of iron on this interaction.

## Material and methods

### Bacterial strains and media

Bacterial strains and plasmids used in the present work are listed in Table 1. The prototypic UPEC strain NU14 was isolated from a patient suffering from cystitis [36]. Bacteria were cultured in Luria-Bertani (LB) medium and nutrient broth (NB) [containing per liter: 8 g nutrient broth (Difco), 5 g NaCl] at 37°C with aeration. NB medium was supplemented with 200 μM α,α’-dipyridyl (Sigma) resulting in NBD medium in order to create iron deplete conditions. Use of antibiotics was provided as necessary (chloramphenicol 20 μg/ml, kanamycin 25 μg/ml, ampicillin 100 μg/ml, tetracycline 12 μg/ml).

**Table 1:** Bacterial strains and plasmids.

Strains and plasmids	Relevant characteristics	Reference
NU14 wt	O18: K1: H7; cystitis isolate	[36]
NU14 ∆*ybtA*	*ybtA* deletion mutant	This study
NU14 ∆*irp2*	*irp2* deletion mutant	This study
NU14 ∆*ybtA* rec	complemented mutant, pWKS30-P*ybtA*; Ap	This study
NU14 ∆*irp2* rec	complemented mutant, pWKS30-P*irp2*; Ap	This study
NU14 *hpi*::Kn	entire *hpi* deleted; Kn	This study
CFT073 wt	O6: K2: H1; urosepsis isolate; Ybt negative	[41]
CFT073 Ybt+	Ybt+, Cm	This study
NU14 3RS	wild type, pMP220-3RS; Tet	This study
NU14 2RS+	wild type, pMP220-2RS+FBS; Tet	This study
NU14 2RS	wild type, pMP220-2RS; Tet	This study
NU14 1RS	wild type, pMP220-1RS; Tet	This study
DH5α	*fhuA2 Δ(argF-lacZ)U169 phoA glnV44 Φ80 Δ(lacZ)M15 gyrA96 recA1 relA1 endA1 thi-1 hsdR17*	Stratagene
DH5α Ybt+	Ybt+, Cm	This study
DH5α 3RS	wild type, pMP220-3RS; Tet	This study
DH5α 2RS+	wild type, pMP220-2RS+FBS; Tet	This study
DH5α 2RS	wild type, pMP220-2RS; Tet	This study
DH5α 1RS	wild type, pMP220-1RS; Tet	This study
**Plasmids**		
pKD3	chloramphenicol template plasmid	[37]
pKD4	kanamycin template plasmid	[37]
pKD46	lambda red recombinase helper plasmid	[37]
pCP20	FLP recombinase helper plasmid	[37]
pWKS30	low-copy plasmid, Ap	[39]
pWKS30-P*ybtA*	expressing YbtA under the control of the native promoter, Ap	This study
pWKS30-P*irp2*	expressing Irp2 under the control of the native promoter, Ap	This study
pCP1	carrying *irp*1-9, *fyuA*, *ybtA* genes and 400 nucleotides of intB, R6K ori; Cm	[42]
pMP220	contains promoterless *lacZ* for transcriptional reporter fusions, Tet	[40]
p3RS	pMP220 carrying entire *ybtA* promoter, Tet	This study
p2RS+	pMP220 carrying two repeated sequences (RS1, RS2) plus Fur binding site, Tet	This study
p2RS	pMP220 carrying two repeated sequences (RS1, RS2) without Fur binding site, Tet	This study
p1RS	pMP220 carrying one repeated sequence (RS2), Tet	This study

### Motility assays

Swimming motility was assessed by using 0.3% LB soft agar plates. A late logarithmic phase culture (OD_600_ = 1.0) was stabbed into the middle of a soft agar plate and incubated at 37°C. Four hours after inoculation motility was quantified every hour by measuring the diameter of swimming bacteria. In order to address the influence of iron excess on motility in a rich medium like LB, soft agar plates were supplemented with different concentrations of additional Fe(III)Cl_3_ (Fluka AG) as indicated. Samples for β-galactosidase assays, western blotting and quantitative real-time PCR were prepared by scraping bacteria off the edge of a motility ring of strains swarming on a 0.5% LB swarm plate. All experiments were performed in duplicates and repeated at least three times. For statistical analysis a paired t-test or the Mann-Whitney U test were performed and results were considered statistically significant if the *p*-value was lower than 0.05.

### Construction of isogenic mutants

Deletion mutants of target genes were generated using the lambda red recombinase approach published by Datsenko and Wanner [37]. Briefly, primers with 40-nt homology extensions to the 5’ and 3’ regions of the gene of interest and 20-nt priming sequences for the template plasmids pKD3 or pKD4 carrying resistance cassettes flanked by FRT recognition target sites were designed (Table 2). The resulting PCR product was then transformed into strains harbouring the helper plasmid pKD46 with lambda red recombinase under an arabinose-inducible promoter. In case of successful replacement of the specific gene, Km^R^ or Cm^R^ transformants were selected. Correct integration of the resistance cassette was confirmed by PCR.

**Table 2:** Oligonucleotides.

Primers for gene disruption		
genes	primers	sequences (5’-3’)
*irp2*	*irp2*.KO.for	CAGCAGTTACATGAAGAGAGCAACCTGATCCAGGCCGGCCTGGAGTGTAGGCTGGAGCTGCTTC
*irp2*	*irp2*.KO.rev	GTTTGAGTTCACGGAGTAATTCGACGCCGGACCAGTGGCGATGCTCATATGAATATCCTCCTTA
*ybtA*	*ybtA*.KO.for	ATGATGGAGTCACCGCAAACGCAATCTGAAATCTCTATTCACCAGTTGGTGGTCGGTGTAGGCTGGAGCTGCTTC
*ybtA*	*ybtA*.KO.rev	CATCCCGCGTTTAAAGGTCGAAGGAGTTACGCCAAACTGTTTCTGGAAGGCGGCACATATGAATATCCTCCTTA
*irp2*	*irp2*.KO.for	CAGCAGTTACATGAAGAGAGCAACCTGATCCAGGCCGGCCTGGAGTGTAGGCTGGAGCTGCTTC
*irp2*	*irp2*.KO.rev	GTTTGAGTTCACGGAGTAATTCGACGCCGGACCAGTGGCGATGCTCATATGAATATCCTCCTTA
*fliI*	*fliI*.KO.for	AGTGTCGCCACTCGCTGGCAAGAACTCTGCCGTCTGGCAGCACCAGGAGTGGTGTAGTGTAGGCTGGAGCTGCTTC
*fliI*	*fliI*.KO.rev	GATCTTTCAGGGTCGCCAGCGCACCATGTTCTGCCATCTGCCGTTATCTCCTGGGCATATGAATATCCTCCTTA
**Primers and probes for TaqMan-PCR**		
**genes**	**primers and probes**	**sequences (5’-3’)**
*16SrRNA*	*16SrRNA*.for	TTGACGTTACCCGCAGAAGAA
*16SrRNA*	*16SrRNA*.rev	GCTTGCACCCTCCGTATTACC
*16SrRNA*	*16SrRNA*.probe	FAM-CGGCTAACTCCGTGCCAGCAGC-TAMRA
*fyuA*	*fyuA*.for	ACACCCGCGAGAAGTTAAATTC
*fyuA*	*fyuA*.rev	AGCGGTGGTATAGCCGGTACT
*fyuA*	*fyuA*.probe	FAM-CCTACGACATGCCGACAATGCCTTATTTAA-TAMRA
*ybtA*	*ybtA*.for	GTTGCCTCTCCTGCCACTTC
*ybtA*	*ybtA*.rev	ATCAGCCAGCAGCAGATCCT
*ybtA*	*ybtA*.probe	FAM-ACCCGATGGAACGCCAGAAACTG-TAMRA
*Irp2*	*Irp2*.for	TGGGTGCCGGGTGAATTA
*Irp2*	*Irp2*.rev	CGTCCGGGAGCGTCAA
*Irp2*	*Irp2*.probe	FAM-ATTTCAACGATCCCCTGCGTAGC-TAMRA
*fliC*	*fliC*.for	CAGGCGATTGCTAACCGTTT
*fliC*	*fliC*.rev	ATACCATCGTTGGCGTTACGT
*fliC*	*fliC*.probe	FAM-TTCTAACATTAAAGGCCTGACTCAGGC-TAMRA
*fliD*	*fliD*.for	GCGTAAGCGCAAGCATCATT
*fliD*	*fliD*.rev	GCCGGTGTCATTTGATGTGA
*fliD*	*fliD*.probe	FAM-ACGTGGGTAACGGTGAATATCGTCT-TAMRA
*flgK*	*flgK*.for	GGGAATAAAACCGCGACGTT
*flgK*	*flgK*.rev	GGAAAGCTGCGTCACCACAT
*flgK*	*flgK*.probe	FAM-AAAACCAGTAGCGCCACGCAAGGT-TAMRA
**Primers for cloning**		
**genes**	**primers**	**sequences (5’-3’)**
*ybtA*	*PybtA*.KpnI.for	TAGCAGggtaccCTGAATTTCCTGATGAATTT
*ybtA*	*ybtA*.PstI.rev	TGCATCctgcagGGCCTCTGTCAGGGAGGAGT
*Irp2*	*Pirp2*.KpnI.for	TAGCACggtaccCCGGGGTCGCGCCCCCCTAA
*Irp2*	*Irp2*.PstI.rev	TCACTActgcagCTATATCCGCCGCTGACGAC
3RS	*PybtA*.KpnI.for	TAGCAGggtaccCTGAATTTCCTGATGAATTT
3RS	*PybtA*.PstI.rev	TCAGCActgcagGACCTGGTTATCTCCCTGTG
2RS+	*PybtA*.2RS+.KpnI.for	TAGCAGggtaccTGGCGTTCTGAGAATTAATG
2RS+	*PybtA*.PstI.rev	TCAGCActgcagGACCTGGTTATCTCCCTGTG
2RS	*PybtA*.2RS.KpnI.for	TAGCAGggtaccAACTCATCTACCCCATTCGG
2RS	*PybtA*.PstI.rev	TCAGCActgcagGACCTGGTTATCTCCCTGTG
1RS	*PybtA*.1RS.KpnI.for	TAGCAGggtaccTATACCCGCATTGGTCTAAG
1RS	*PybtA*.PstI.rev	TCAGCActgcagGACCTGGTTATCTCCCTGTG

### Cloning and recombinant DNA techniques

Standard genetic methods were performed mainly as described by Sambrook and Russell [38]. Enzymes were purchased from Fermentas and used according to the manufacturer’s recommendations. Primers and plasmids used in this study are listed in Table 2. Plasmids for complementation of the deleted genes were constructed by PCR amplification of the wild type alleles under the control of their own promoter. The PCR products were purified using a QIAquick PCR purification kit (Qiagen), digested with *KpnI* and *PstI* and cloned into low-copy plasmid pWKS30 [39]. In order to generate transcriptional reporter gene fusions different fragments of the *ybtA* promoter were PCR amplified, digested with *KpnI* and *PstI* and fused with a promoterless *lacZ* gene in plasmid pMP220 [40]. The iron independent YbtA expression was achieved by cloning *ybtA* into plasmid pWKS30 under the control of a *lac* promoter using *KpnI* and *PstI* as restriction enzymes. *YbtA* under the control of a *lac* promoter from plasmid pWKS30-ybtA was PCR amplified, digested with *BamHI* and *SalI* and cloned into the tetracycline cassette of plasmid pACYC184. A yersiniabactin complemented derivative of the wild type strain CFT073 [41] was constructed by integrating the functional *hpi* core region of *Y*. *enterocolitica* using plasmid pCP1 [42]. All genetic constructs established in this study were validated by screening PCRs before they were used in experiments.

### β-galactosidase assays

β-galactosidase activities of reporter gene fusions were quantified mainly according to standard procedures [43] and expressed as miller units. In order to focus specifically on the regulatory effect of YbtA on its own promoter, a second plasmid pWKS30-*ybtA* expressing YbtA under the control of a *lac* promoter was introduced. All experiments were performed in duplicates and repeated at least three times. For statistical analysis a paired t-test or the Mann-Whitney U test were performed and results were considered statistically significant if the *p*-value was lower than 0.05.

### Western blotting

For the detection of flagellar expression bacteria swarming on 0.5% LB swarm plates were scraped off carefully, resuspended in PBS and finally adjusted to an OD_600_ = 1.0. Bacteria from this standardized culture were pelleted by centrifugation and subjected to sodium dodecyl sulfate-polyacrylamide gel electrophoresis (SDS-PAGE) and subsequently transferred to a Protran® nitrocellulose transfer membrane (Whatman). Rabbit polyclonal antiserum to H7 flagellin (kind gift of B. Westerlund-Wikström) was used as primary antibody. For the detection of high pathogenicity island proteins bacterial cultures grown in LB medium or NBD medium were collected, adjusted to an OD_600_ = 1.0 and finally analysed using rabbit polyclonal antiserum to YbtA (kind gift of A. Rakin) and FyuA [44]. The respective samples were loaded on a SDS-PAGE and stained with Coomassie-Brilliant-Blue, which served as loading control.

### RNA extraction and quantitative real-time PCR (TaqMan)

Different RNA samples were prepared as described above. RNA extraction was performed using the Trizol (Invitrogen) method [45]. Total RNA was first treated with DNase I (Fermentas) to remove contaminating genomic DNA. Then, first-strand cDNA was synthesized using random hexamers and RevertAid H Minus M-MuLV Reverse Transcriptase (Fermentas) according to the manufacturer’s recommendation. TaqMan PCR was run on a 7500 Fast Real-Time PCR System (Applied Biosystems). Primers were designed using the Primer Express software (Version 3.0, Applied Biosystems) and probes were labelled with FAM at the 5’-terminus and TAMRA at the 3’-terminus (Table 2). TaqMan PCR reactions were carried out in a final volume of 25 μl containing TaqMan Gene Expression Master Mix (Applied Biosystems), primers, probe and 30ng of cDNA. Transcript levels were normalized to 16 S rRNA. Data were analyzed by the 2^-∆∆^ method as described by Livak and Schmittgen [46].

## Results

### The influence of yersiniabactin synthesis on flagellar motility in UPEC

**Fig 1:**
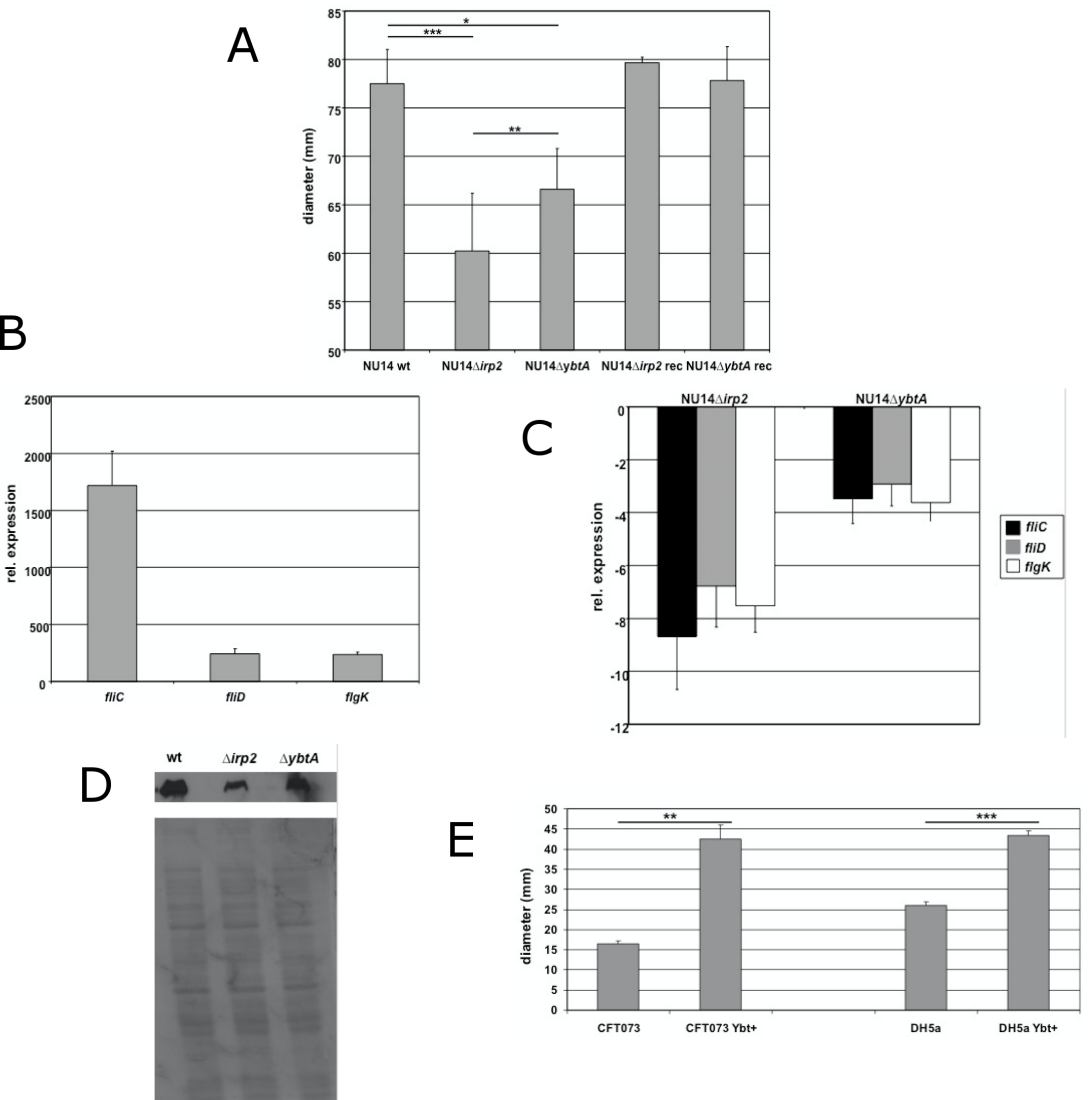
In vitro characterizations of the contribution of the HPI to motility in UPEC strain NU14. (A) Deletions of both *irp2* and *ybtA* resulted in the reduction of the motility diameter on LB soft agar plates (0.3%). Motility was restored in complemented strains. qPCR for flagellar genes during motility in wild type strain NU14 (B) and in HPI mutants (C). Data were normalized to 16S rRNA. Bacteria rotating in LB at 37°C were used as the calibrator for (B) and wild type strain NU14 during motility served as the calibrator for (C). (D) Detection of flagellin expression using H7 antiserum. Lane 1, NU14 wild type; lane 2, NU14 Δ*irp2*; lane 3, NU14 Δ*ybtA*. (E) The impact of yersiniabactin production on motility in yersiniabactin-negative strains. CFT073 ybt+ and DH5α ybt+ displayed increased motility compared to the respective wild type strains. All experiments were performed in triplicates and repeated at least three times. Error bars represent standard deviations. *p<0.05, **p<0.01, ***p<0.001.

In a first attempt to determine the role of the HPI in flagellum-mediated motility, defined mutations affecting genes involved in the biosynthesis of yersiniabactin were generated in the prototypic UPEC strain NU14. *Irp2*, coding for HMWP2 (high molecular weight protein 2) [47], and *ybtA*, encoding a transcriptional regulator of the AraC-like family [19], were deleted according to the protocol described by Datsenko and Wanner [37]. The functional impact of these deletions on yersiniabactin production was verified in a yersiniabactin detection *luc* reporter assay (data not shown). Motility was first assessed performing swimming assays on LB soft agar plates (**Fig 1A**). Although LB is considered to be a rich medium with excess of iron available, measuring of the diameter of swimming bacteria revealed a reduction of motility compared to the wild type strain. After 7 hours of incubation the average motility diameters of the *irp2* mutant (60.25 mm ± 5.93 mm; *p*<0.001) and the *ybtA* mutant (66.58 mm ± 4.21 mm; *p*<0.05) were both significantly decreased compared to the wild type strain (77.50 mm ± 3.55 mm), with the deletion of *irp2* resulting in a stronger impairment than the *ybtA* mutant (*p*<0.01). The complementation of both *irp2* and *ybtA in trans* restored the motility to wild type levels, thus confirming that the phenotype was due to the deletion of *irp2* and *ybtA*. In order to investigate this decrease in motility, we performed qPCR to determine whether the phenotype is regulated at the transcriptional level. *FliC*, *fliD* and *flgK* genes coding for flagellin, a filament-capping protein and a hook-filament junction protein, respectively, are class III flagellar genes, which were selected to study gene expression [48]. Performing motility assays, flagellar genes displayed a strong induction relative to the wild type strain cultivated in LB medium at 37°C (**Fig 1B**). *FliC* was the most upregulated gene with a 1700-fold increase in gene expression. The amounts of *fliD* and *flgK* transcripts were increased 245-fold and 235-fold, respectively. This result confirmed the strong induction of flagellar genes at the transcriptional level. Next, we tested different HPI mutants and compared their gene expression with the wild type strain (**Fig 1C**). We observed a downregulation of the studied flagellar genes in all HPI mutants. NU14 ∆*irp2* demonstrated the strongest decrease in transcription of *fliC*, *fliD* and *flgK*, down 8.7-fold, 6.8-fold and 7.5-fold, respectively, relative to the wild type strain. Transcriptional analysis indicated that the abolishment of yersiniabactin production, as represented by the *irp2* mutant, resulted in decreased flagellin expression. Therefore, we investigated the production of flagella in different HPI mutants using H7 flagellin antiserum (**Fig 1D**). The reduced gene expression of *fliC* in the *irp2* mutant correlated with a decrease in flagellin expression. These data clearly demonstrated that the ability to produce yersiniabactin promotes flagella expression and motility in ExPEC. This finding prompted us to investigate whether the gain of yersiniabactin biosynthesis in a yersiniabactin deficient strain would also affect motility. UPEC strain CFT073 was isolated from a patient with acute pyelonephritis [41] and represents a natural yersiniabactin-deficient strain due to mutations within essential biosynthetic genes. We restored yersiniabactin synthesis in CFT073 by integrating the functional HPI core region of *Y*. *enterocolitica* [42] creating strain CFT073 Ybt+. Functionality was confirmed in a yersiniabactin detection reporter assay (data not shown). We performed motility assays with both CFT073 wild type and CFT073 Ybt+ and we observed a significant increase in the motility of the yersiniabactin producing derivative CFT073 Ybt+ **(Fig 1E).** After 9 hours of incubation on a LB soft agar plate the diameter of CFT073 Ybt+ was 42.5 mm (± 3.5 mm) compared to 16 mm (± 2 mm) (*p*<0.01). Even the equipment of K12 *E*. *coli* strain DH5α, which is an HPI-negative strain, with a functional HPI promotes motility **(Fig 1E)**. After 12 hours of incubation the motility diameter of DH5α increased from 26 mm (± 1 mm) to 43 mm (± 0.87 mm) due to additional production of yersiniabactin (*p*<0.001). These results support the contribution of yersiniabactin production to motility.

### The loss of flagellin expression does not affect the functionality of the HPI

**Fig 2:**
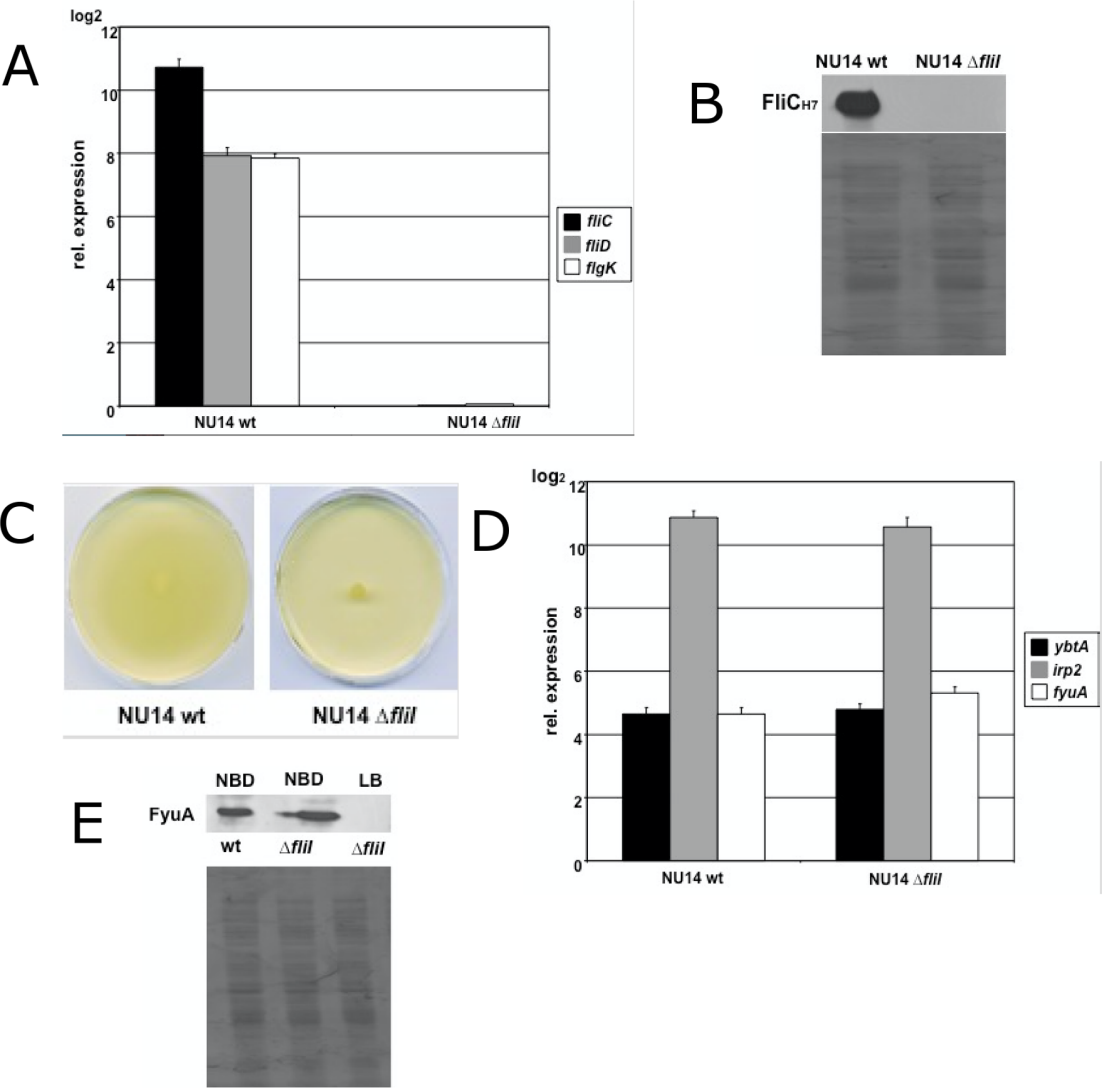
The loss of flagellar expression does not influence the activity of the HPI under iron deplete condition. (A) qPCR for flagellar genes during motility in strains NU14 wild type and flagellar mutant NU14 Δ*fliI*. Deletion of *fliI* resulted in the loss of flagellar gene expression of *fliC*, *fliD* and *flgK*. Data were normalized to 16S rRNA. Bacteria shaken in LB at 37°C were used as the calibrator. (B) Detection of flagellin expression using H7 antiserum. Flagellar mutant NU14 Δ*fliI* showed no expression of flagellin. (C) Mutant strain NU14 Δ*fliI* was non-motile on LB soft agar plates. (D) qPCR for HPI genes of NU14 wild type and NU14 Δ*fliI* under iron deplete condition. Transcription rates for *ybtA*, *irp2* and *fyuA* of the flagellar mutant NU14 Δ*fliI* displayed no difference relative to the wild type strain NU14 under iron restriction. (E) Detection of FyuA expression in NBD medium. No difference in FyuA expression could be detected between the wild type strain and the flagellar mutant. NU14 Δ*fliI* showed no expression of FyuA under iron rich conditions in LB medium.

Since the loss of yersiniabactin production resulted in reduced motility, we further addressed the question whether the disruption of flagellar biosynthesis, leading to a non-motile phenotype, would also affect the ability to synthesize yersiniabactin. We created a flagellar mutant by deleting *fliI*, a class II gene, which codes for an ATPase involved in type III flagellar export [48]. This mutant showed neither induction of flagellar gene expression **(Fig 2A)** nor detectable expression of flagellin **(Fig 2B)**, and consequently, the mutant was non-motile on LB soft agar plates **(Fig 2C).** The cultivation of the flagellar mutant under iron deplete conditions (**Fig 2D)** had no adverse effect on the transcript levels of the HPI genes *ybtA*, *irp2* and *fyuA* (*p*>0.05). Also the detection of FyuA expression by western blot analysis of the *fliI* mutant under iron limited cultivation in NBD medium showed no difference compared to the wild type strain (**Fig 2E)**. Therefore, these data demonstrated that the impairment of the flagellar system does not influence the function of the HPI.

### Stimulation of the HPI with additional iron under iron abundance promotes motility

**Fig 3:**
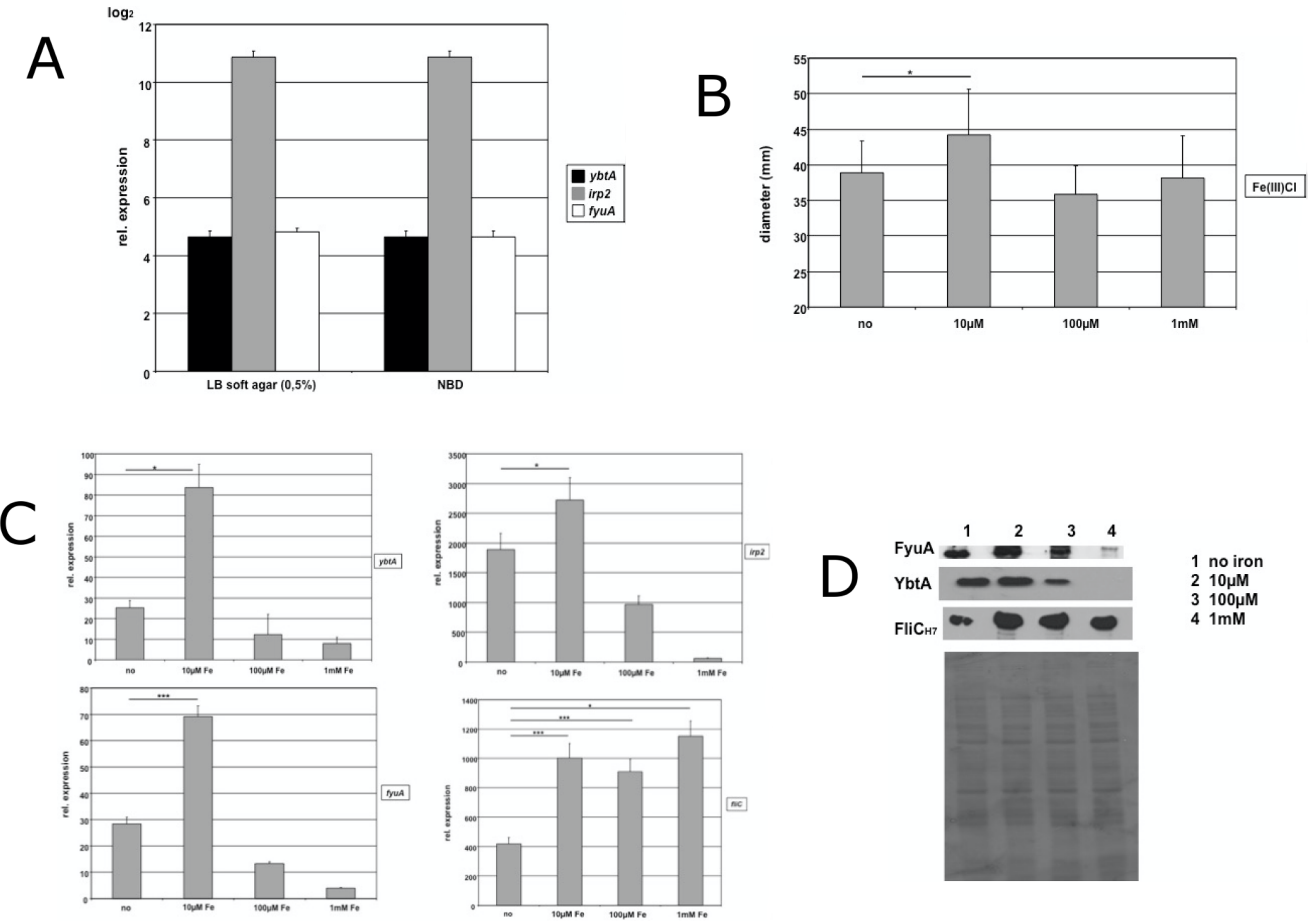
The abundance of iron stimulates the HPI during motility. (A) qPCR for the HPI genes *ybtA*, *irp2* and *fyuA* in NU14 wild type strain showed equal transcription rates for both iron depletion in NBD and during motility on LB soft agar plates. (B) The supplementation of 0.3% LB soft agar plates with 10 μm Fe(III)Cl_3_ resulted in increased motility (*p<0.05). The addition of 100 μM or 1 mM Fe(III)Cl_3_ did not affect motility diameters significantly. (C) qPCR of swarming NU14 on LB soft agar plates supplemented with different concentrations of Fe(III)Cl_3_. Only addition of 10 μm Fe(III)Cl_3_ led to the increased gene expression of the HPI genes *ybtA*, *irp2* and *fyuA*. The transcription of *fliC* was stronger upregulated during motility with the addition of iron, but displayed no difference for various concentration of Fe(III)Cl_3_. (D) Western blot analysis of the expression of YbtA, FyuA and flagellin on LB soft agar plates supplemented with different concentrations of iron. YbtA and FyuA expression was only increased for 10 μM Fe(III)Cl_3_. The addition of 1 mM ferric iron repressed almost completely expression of YbtA and FyuA. Rising concentrations of iron led to an overall enhanced expression of flagellin with no difference for various concentrations.

Iron uptake systems are essential to the overall fitness of pathogens and they are supposed to be activated only by scarcity of iron [49]. Strikingly, the HPI contributes to motility under iron rich conditions. Our first step to investigate this phenomenon was to perform transcriptional analysis of the UPEC strain NU14 cultivated in iron deplete medium (NBD medium) and swarming on a LB soft agar plate **(Fig 3A)**. In NBD medium the gene expression of *ybtA*, *irp2* and *fyuA* was strongly induced. Surprisingly, the transcription rate of swarming NU14 showed no significant difference (*p*>0.05), indicating the strong activation of the HPI during motility. This strong induction of transcription prompted us to study whether additional iron may suppress this activation during motility or stimulate even stronger. For that purpose, we supplemented LB soft agar plates with different concentrations of Fe(III)Cl_3_. We applied 10 μM, 100 μM and 1 mM of ferric iron. Motility assays indicated a significant increase only for 10 μM Fe(III)Cl_3_ compared to standard LB soft agar plates without any additional iron (*p*<0.05) **(Fig 3B)**. Higher concentrations appeared to mediate no relevant benefit for motility. Real-time PCR was further carried out to analyse the gene expression of both the HPI and the flagellar system (**Fig 3C**). In the case of supplementation with 10 μM of ferric iron we observed a stronger induction of genes of the HPI. *YbtA* increased from 25-fold to 84-fold induction (*p*<0.05), *irp2* showed a rise from 1800-fold to 2800-fold (*p*<0.05), and *fyuA* was upregulated from 28-fold to 70-fold (*p*<0.001). Using higher concentrations of iron we observed a suppression of transcript levels, which finally resulted in the inactivation of the HPI at 1 mM of FeCl_3_. The transcription of *fliC* could be stimulated with additional iron, but displayed no difference for the various concentrations of iron used. Corresponding results could be obtained by western blotting **(Fig 3D)**. The expression of both YbtA and FyuA increased when 10 μM of ferric iron were added, and diminished at higher concentrations until no expression was detectable. The investigation of flagellin production revealed a rise in expression with additional iron, but according to the qPCR data we could not observe significant changes for different concentrations of FeCl_3_. At this point, our data clearly prove that the HPI is active during motility under iron rich conditions, and the HPI is even stronger activated when 10 μM of ferric iron is added, instead of being repressed by this excess of iron.

### Activation of the *ybtA* promoter during motility

**Fig 4:**
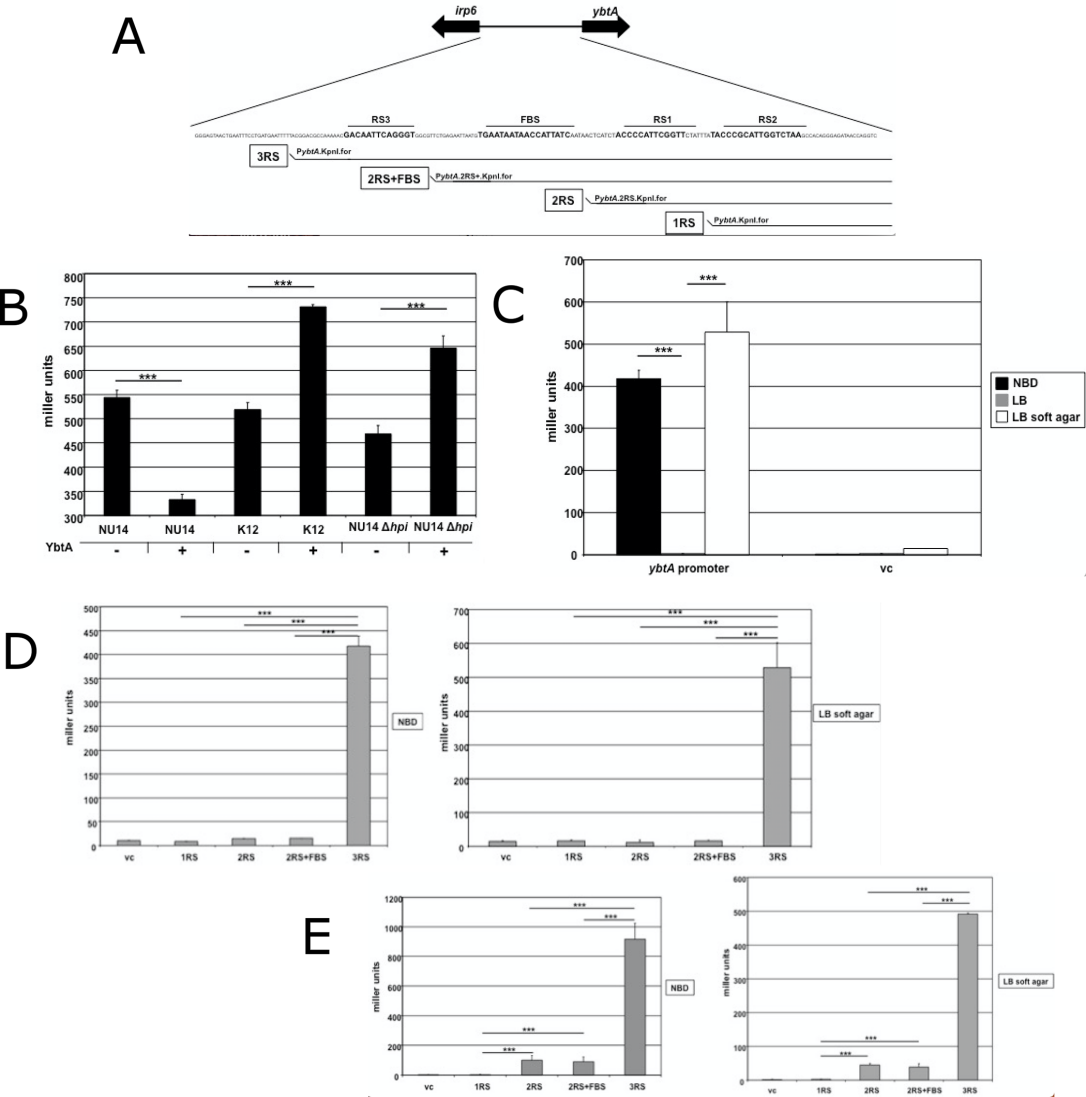
Analysis of the *ybtA* promoter activity under iron depletion and during motility. (A) Construction of transcriptional *lacZ* reporter fusions with different fragments of the *ybtA* promoter region. (B) Influence of the YbtA expression on the *ybtA* promoter activity under iron depletion. The additional expression of YbtA showed a decrease in miller units compared to the empty vector control in NU14 wild type (***p<0.001), but increased significantly the reporter signal in the HPI-negative strains DH5α and NU14 Δ*hpi*, respectively (***p<0.001). (C) The reporter activity of NU14 wild type strain carrying p3RS cultivated in NBD medium, LB medium and during motility on LB soft agar plates. Elevated miller units could be detected only under iron scarcity and during motility (***p<0.001). The reporter activity for different fragments of the *ybtA* promoter region under iron restriction and during motility in UPEC strain NU14 (D) and K12 *E*. *coli* DH5α (E). In both backgrounds the highest promoter activity was only detected with construct p3RS for both growth in NBD medium and during motility on LB soft agar plates.

The YbtA-mediated transcriptional regulation of genes like *irp2*, *irp6* and *fyuA* has been elucidated in previous studies [17; 18], which clearly highlighted the essential role of YbtA. The absence of this transcriptional regulator results in a negligible activity of the HPI. But data acquired so far still left a few questions unanswered. With the induction of *ybtA* transcription, representing the starting point for activation of the HPI, our first step was to construct several transcriptional *lacZ* reporter fusions with different fragments of the *ybtA* promoter region **(Fig 4A)**. We fused the entire promoter including all three YbtA binding sites, termed repeated sequences (RS), and the Fur box to the *lacZ* gene to confirm the published data concerning *ybtA* regulation. YbtA is supposed to activate the transcription of *irp2*, *irp6* and *fyuA* and represses its own transcription [17–19]. In order to verify the auto-repression of *ybtA* we introduced both plasmid p3RS and plasmid pWKS30-*ybtA* into UPEC strain NU14. PWKS30-*ybtA* contains *ybtA* under the control of a *lac* promoter, so iron independent expression of YbtA is achieved. As depicted in **Fig 4B,** NU14 with additional YbtA expression showed a reduced reporter activity of 330 miller units compared to 540 miller units (*p*<0.01) in the strain carrying the empty vector control. We wanted to know whether this expected phenotype could be reproduced in an HPI-negative background, like in K12 *E*. *coli* DH5α. This setup actually allows the exclusive analysis of the interaction of YbtA with its own promoter, because the influence of all known and still unknown *cis* regulatory elements on the HPI can be excluded. Interestingly, in DH5α the additional expression of YbtA led to an increase in reporter activity of 41% relative to the strain without YbtA expression (*p*<0.001). This surprising observation prompted us to study this effect in the UPEC strain NU14 ∆*hpi*, where the entire HPI has been deleted. Measuring of miller units in the mutant NU14 ∆*hpi* revealed that the extra expression of YbtA indeed induced the reporter activity. We observed an almost 40% increase of miller units when additional YbtA was expressed (*p*<0.001). After all, these promoter studies suggest that YbtA itself activates its own transcription, at least in an HPI-negative genomic background.

We kept on investigating the role of the *ybtA* promoter region in flagellum-mediated motility. The activation of the HPI starts always with the induction of *ybtA* transcription. So we fused different parts of the *ybtA* promoter region to the *lacZ* gene and analyzed the reporter activities of motile bacteria (**Fig 4A**). The constructs comprised a selection of reporter fusions including the whole promoter region (p3RS), two YbtA binding sites plus the Fur box (p2RS+), two YbtA binding sites without the Fur box (p2RS), only one YbtA binding site (p1RS) and finally a short fragment of the promoter region without any known binding site. Initially, we compared promoter activities in UPEC strain NU14 using reporter plasmid p3RS under different conditions, i.e. cultivation in NBD medium, cultivation in LB medium and swarming NU14 (**Fig 4C**). As expected, iron depletion activated the *ybtA* promoter compared to the cultivation under iron excess. Consistent with the data presented above, the reporter activity was highly elevated during swarming. Approximately 420 miller units (± 20.1) for bacteria in NBD medium and 530 miller units (± 70.2) for swarming bacteria confirmed, that the HPI is almost equally activated. To determine the role of single promoter fragments containing different regulatory elements, we introduced various reporter constructs into the NU14 wild type strain and compared the reporter signals of bacteria grown under iron starvation with motile bacteria (**Fig 4D**). The resulting data demonstrated that the entire promoter region with all three YbtA binding sites is necessary to fully activate the *ybtA* promoter. Only the reporter plasmid p3RS displayed a strong signal of 417 miller units (± 20.9). When the swarming UPEC NU14 was examined, we were able to observe the same pattern. Only the wild type strain carrying the reporter construct p3RS including the entire promoter region showed an elevated reporter activity of 528 miller units (± 73.5). To investigate if the observed pattern in UPEC strain NU14 also accounted for a HPI negative strain, reporter activities were detected in K12 *E*. *coli* strain DH5α carrying also the same reporter constructs (**Fig 4E).** The cultivation in NBD medium resulted in a strong signal of 490 miller units (± 3.3) for reporter plasmid p3RS. Interestingly, we observed a slight reporter activity for constructs p2RS+ and p2RS, with both containing two YbtA binding sites. Once again, the pattern of reporter activity was identical to the results acquired under iron depletion. Plasmid p3RS displayed the highest rate with 918 miller units (± 100.6), whereas p2RS+ and p2RS produced weak signals. Overall, the promoter studies clearly show that the entire promoter region including all YbtA binding sites is mandatory for complete promoter activity. Furthermore, the *lacZ* reporter activity patterns during motility were similar in the HPI-positive strain NU14 and the HPI-negative strain DH5α suggesting a common mechanism of induction for the HPI, which is independent of YbtA and yersiniabactin production.

## Discussion

Numerous studies have been published over the last decades highlighting the fundamental role of iron uptake systems for the overall fitness of pathogenic bacteria [10; 50–53]. The efficient colonization of the urinary tract by UPEC requires the coordination of a plethora of different iron acquisition strategies in order to satisfy the increased need for iron in this hostile environment. Especially the HPI turned out to be essential for the virulence potential of many different pathogens [8–10]. The objective of this present work was to investigate a possible connection between iron acquisition as accomplished by the HPI and motility in UPEC strain NU14. The first results clearly demonstrated that mutations of *ybtA* and *irp2* resulting in the impairment of yersiniabactin production affected flagellar motility. The most interesting aspect of this observation is that motility assays were performed on LB soft agar plates, with LB representing an iron rich condition. External iron excess is supposed to repress transcription of genes involved in iron acquisition through the action of the ferric uptake regulator Fur [49]. However, transcriptional and translational data of HPI mutants motile on LB soft agar plates provided evidence that the reduction of motility is due to a decrease in both transcript levels of flagellar genes and production of flagellin. The gain of the ability to synthesize yersiniabactin improved the motility in pathogenic and non-pathogenic *E*. *coli* strains. These results prove that a functional HPI promotes flagellum-mediated motility. We investigated whether this interaction between HPI and flagellar system is mutual. The disruption of flagella expression had no effect on the activity of the HPI under iron deplete cultivation. It appears that induction of flagellum-mediated motility constitutes a stimulus that activates the HPI regardless of the external content of iron. This unexpected phenomenon prompted us to investigate how different concentrations of iron might affect this interaction. Most strikingly, during motility additional iron induced even more both motility and the HPI. Beside the activation of the HPI under iron excess, the further stimulation of a siderophore system with higher amounts of iron under iron excess is quite astonishing. To our knowledge, this is the first report of a higher regulatory mechanism of iron homeostasis that extends beyond the regulation by Fur and external iron content. The cultivation in an iron rich medium like LB shows no induction of iron uptake systems due to the binding of Fur to Fur boxes upstream of iron regulated genes [49]. But as soon as the motility is assessed on LB soft agar plates, UPEC strain NU14 showed a strong activation of the HPI that can even be maximized. With regard to Fur, it could be possible that the ferric uptake regulator itself might be affected or an additional factor prevents binding of Fur to the Fur binding site. Using *lacZ* transcriptional reporter fusions we analysed the *ybtA* promoter activity under different conditions to identify elements of the promoter region necessary for targeting the HPI during motility. We confirm previously published data [17] that all 3 YbtA binding sites are required for full promoter activity under iron scarcity. This also accounts for motility. One surprising finding concerning *ybtA* regulation was the unexpected stimulation of the *ybtA* promoter by YbtA itself. Published reports provided evidence for auto-repression [17; 19], but the HPI background of tested strains was always present in the respective studies. The effect of self-inhibition was confirmed in our experiments when we performed reporter assays in a HPI-positive strain, even in strains with different HPI mutations (data not shown). The exclusive investigation of the influence of YbtA on its own promoter requires the complete deletion of the HPI. In that way, unknown putative *cis*-regulatory elements located on the HPI can be excluded. In an HPI-free background we were able to detect activation of the *ybtA* promoter *lacZ* fusion with the additional expression of YbtA for both pathogenic and non-pathogenic strains. This is not the only interesting observation. It is important to stress, that the binding of YbtA to its promoter can occur in the absence of yersiniabactin. The results for K12 *E*. *coli* DH5α carrying the YbtA expressing plasmid demonstrated the yersiniabactin-independent interaction of YbtA with its promoter. These new insights complement the current understanding of YbtA-mediated regulation of the HPI. The initial induction of the HPI starts with transcription and translation of YbtA. Small amounts of this regulator will stimulate stronger the expression of YbtA, so that all the other YbtA-dependent operons can be transcribed. This will start the production of yersiniabactin until the HPI is fully activated. At this point, further expression of YbtA may be damped. The molecular mechanism of this inhibition cannot be explained with our experimental set up. But DNA bending mechanisms stabilized through YbtA with or without yersiniabactin, as it is known for the AraC-mediated regulation, may be possible [54]. Further experiments are needed to address this interesting regulatory aspect. As far as the newly discovered HPI-flagella interaction is concerned, the selection of various *ybtA* promoter fragments fused to the *lacZ* reporter gene allowed to examine whether the promoter activity of *ybtA* under iron deplete conditions was comparable to the process of motility. Strikingly, maximum promoter activity was only detected for the entire promoter region of the reporter construct p3RS. This was the case for both UPEC strain NU14 and laboratory strain DH5α. The full activity under iron restriction might be explained by the inactivation of Fur, but the results of motility assays under iron rich conditions point towards a different mechanism. Fur is supposed to inhibit transcription because of iron abundance. However, a strong signal was measured in both strains. Reporter constructs p2RS+ and p2RS, with a Fur box only included in p2RS+, indicated that Fur might not be the missing link of the flagella-HPI interaction since there is no difference in promoter activity between both reporter plasmids. It is remarkable for the laboratory strain DH5α that neither YbtA nor yersiniabactin can be involved in *ybtA* promoter activity, but there is still a strong signal detectable. Due to the presence of a Fur box in p3RS and p2RS+, the role of Fur for this phenomenon seems to be negligible, otherwise plasmid p2RS+ is supposed to display the same activity. The possibility of an unknown factor, maybe another regulator of the AraC-like family, binding to all YbtA binding sites in YbtA-like fashion may be a theory to explain this observation. This hypothesis might also account for the induction of YbtA expression during motility. In both strains NU14 and DH5α, an unknown factor acting alone or in complex with additional elements seems to occupy the entire promoter region in order to activate YbtA expression. It is unclear, whether this missing link is encoded by the *E*. *coli* core genome, so the same factor is responsible, or whether the mediating factors differ in the strains NU14 and DH5α.

Overall, the data presented in this study clearly showed how the flagellar system activates the HPI by addressing the *ybtA* promoter region. The evasion of important regulatory mechanisms of iron homeostasis seems to be counterbalanced to optimize motility. This work highlights the multi-functional role of the HPI and promotes the concept of a complex but orchestrated network of virulence and fitness factors supporting ExPEC to develop into this successful pathogen.
